# Using Community Conversations to explore animal welfare perceptions and practices of rural households in Ethiopia

**DOI:** 10.3389/fvets.2022.980192

**Published:** 2022-09-26

**Authors:** Mamusha Lemma, Rebecca Doyle, Gezahegn Alemayehu, Mesfin Mekonnen, Adem Kumbe, Barbara Wieland

**Affiliations:** ^1^Animal and Human Health Program, International Livestock Research Institute, Addis Ababa, Ethiopia; ^2^Royal (Dick) School of Veterinary Studies, University of Edinburgh, Edinburgh, United Kingdom; ^3^Oromia Agricultural Research Institute, Yabello Pastoral and Dryland Agriculture Research Centre, Yabello, Ethiopia; ^4^Institute of Virology and Immunology, Mittelhäusern, Switzerland; ^5^Department of Infectious Diseases and Pathobiology, Vetsuisse Faculty, University of Bern, Bern, Switzerland

**Keywords:** animal welfare, human-animal relationships, smallholder production, Community Conversations, Ethiopia

## Abstract

There is a scarcity of data on animal welfare and its impact on livelihoods to inform animal welfare initiatives in Ethiopia. Perceptions and practices of rural households toward animal welfare are influenced by socio-cultural, demographic, and agroecological factors. We conducted Community Conversations in two geographically and culturally diverse regions of Ethiopia to explore the attitudes and practices of rural households regarding animal welfare and its impact on livelihoods. Community Conversations are facilitated dialogues among rural households to explore their perceptions, practices, constraints, and needs and identify and co-create solutions to improve the welfare of their animals. We used single- and mixed-sex discussion groups to understand community members' gendered perceptions of animal welfare and influence their attitudes and practices toward gender-equitable roles in animal welfare management. In the Community Conversations, community members readily described the biological needs of their animals but there was also a good acknowledgment of the behavioral and affective state needs of animals. Identified constraints for animal welfare included feed and water shortage, limited veterinary support, and poor animal handling practices. Community members described the welfare of their animals as being intertwined with their own livelihoods and identified productive, public health, and non-economic benefits of good animal welfare. Raising awareness of animal welfare within rural communities through Community Conversations is a useful way to both identify livestock production needs as well as engage community members in making practical improvements in animal welfare. The understanding of perceptions, practices, and needs of rural households in animal welfare helps engage communities in starting behavioral change and provides insights into developing context-specific welfare improvement interventions. Community Conversations are also an effective way to feedback community voices into planning to build a bottom-up implementation of animal welfare programs.

## Introduction

The lives of animals and people are inextricably linked. Scientific research on animal welfare has predominantly concentrated on intensive production systems in the industrialized world (1). Research on animal welfare has been induced by public concerns over the welfare of animals kept in confinement production systems (2, 3). The concern about animal welfare has tended to emphasize different components of animal welfare. An integrated concept of animal welfare comprises the physical health and biological functioning of animals (such as freedom from disease, injury, and hunger), affective states of animals (like pain, distress, and pleasure), and the ability of animals to live reasonably natural lives by carrying out natural behavior and having natural elements in their environment (4).

The rising public and scientific concern regarding animal welfare has increased demands on governments and organizations worldwide to adopt animal welfare policies, legislations and regulations and create public awareness (5). More recently, there is also a growing body of literature focusing on public concerns and farmers' attitudes toward animal health and welfare (6).

While animal welfare has been a concern of developed countries for many decades, it has recently also gained more attention in low- and middle-income countries (LMICs) (7). It has become an important factor in trade in animal products and a concern for food safety and public health. For developing countries, like Ethiopia, to access global markets, it is crucial that international animal welfare and food safety standards are established and observed (8).

Animal welfare also contributes to the achievement of the United Nations Sustainable Development Goals (SDGs) and promotes the One Health approach. Caring for animals is a pathway to improving both human and animal wellbeing (9). Therefore, the disregard for animal welfare translates into negative impacts on human welfare as the welfare of human beings and animals is inextricably linked.

In Ethiopia, smallholder farmers and pastoralists play a key role in animal management and welfare (10). However, relatively little is known about animal health and welfare-related issues farmers and pastoralists find important and how that translates into good husbandry practices (11). There is a scarcity of data on how smallholder farmers and pastoralists perceive animal welfare, what their practices are, and their understanding of the relationship between animal welfare, productivity, and livelihoods (12). Previous animal welfare studies in Ethiopia have mainly focused on animal transport and slaughter (13), but little has been done at the level of farmers and pastoralists. While the big problems are occurring during transport, the problems should nevertheless not be ignored at the farm level where the animals spend most of their lives.

Using Community Conversations, this study contributes to the body of scientific knowledge on animal welfare by exploring the perceptions, constraints, needs, and practices of smallholder farmers and pastoralists in animal welfare in a developing country context. Community Conversations are powerful tools to engage community members in collaborative learning, reflection, and problem-solving, and facilitate community outreach through social learning and peer influence (14). The practical purpose of the study is to improve the welfare of animals and humans by changing the attitudes and practices of animal owners in developing countries.

## Materials and methods

### Description of the study sites

In October 2019, Community Conversations on animal welfare and livelihoods were conducted in two rural communities in Ethiopia: Darito community in Yabello district of Oromia region, and Sine Amba community in Menz Gera district of Amhara region. These were sites where the Consultative Group on International Agricultural Research Program on Livestock (CRP Livestock) implemented livestock research interventions to improve the livelihoods of smallholder livestock producers. The sites were selected based on their livestock density, agroecology, and agricultural production systems.

The study sites are linguistically, culturally, and agro-ecologically diverse. The population in the Menz Gera site dominantly follows Orthodox Christianity and belongs to the Amhara ethnic group. The population in the Yabello district belongs to the Oromo ethnic group and the majority of the Borana people are Muslim although some practice traditional religion. The agroecology and production system characteristics of the study sites are shown in Table 1. Livestock production in Ethiopia is broadly classified into pastoral, agro-pastoral, and mixed crop-livestock production systems. With an altitude of 2,800–3,100 meters above sea level (masl), the topography of the Menz Gera district consists of plain, mountain, gorge, and undulated land features. The district has bimodal annual rainfall between 900 and 1,000 mm with a mean annual temperature of 12°C. The agricultural production system of the Menz Gera district is a highland mixed crop-livestock production system dominated by crops (15). Livestock production, especially cattle and small ruminants, remains the main source of livelihood for the population.

**Table 1:** Description of the study sites.

**Region**	**District**	**Community**	**Agroecology**	**Production system**	**Altitude (m)**	**Rainfall (mm)**	**Temperature (^0^C)**
Oromia	Yabello	Darito	Dry lowland	Mixed crop-livestock	1,800	500	24
Amhara	Menz Gera	Sine Amba	Moist highland	Mixed crop-livestock	3,100	900–1,000	12

Yabello district is classified as arid and semi-arid rangelands, with pockets of sub-humid zones. The rangelands are dominated by savanna vegetation, with varying proportions of open grasslands consisting of perennial herbaceous and woody vegetation (16). The district has a pastoralism and agro-pastoral production system dominated by livestock production which remains the main source of food, income, and social prestige. Livestock husbandry in lowland agroecology is dominated by goats, cattle, sheep, and camels. With an altitude of 350–1,800 masl, the Yabello district has bimodal rainfall. The mean annual rainfall is 500 mm with considerable inter-annual variability and the mean annual temperature is 24°C (17).

In the study sites, livestock forms an important part of the livelihoods of the communities (10). Feed and water shortages and animal diseases are the major livestock production constraints (18, 19). Women and men play different roles in livestock management (20). Women are commonly involved in feeding animals, cleaning barns, caring for small and sick animals, and milking cows. Men are responsible for gathering or purchasing animal feed and herding and watering animals in distant locations. However, gender norms and practices as well as the weak gender capacity of service providers limit women's access to and use of livestock services including information and training (21).

### The Community Conversation approach and process

Community Conversations are participatory engagement and learning processes where community members and local service providers work with trained facilitators to collectively identify community strengths and constraints, analyze community values and practices, and explore strategies for addressing livestock management challenges (22, 23). They encourage critical discussions and reflections among community members and local service providers on pertinent livestock development issues leading to the development of community actions to make desired improvements. The Community Conversations approach has its roots in participatory approaches such as social learning theory (24), actor-oriented approach (25), participatory learning and action (26), and participatory action research (27).

Drawing upon principles and practices of these participatory approaches, we designed Community Conversations protocol (28). The protocol provides methodological guidance and process steps for the implementation and documentation of Community Conversations. The approach has already been tested and documented in the CRP Livestock in Ethiopia addressing different livestock management issues (14, 23). It involves iterative learning, action and reflection steps: (1) exploration and analysis of existing community knowledge and practices; (2) introduction of new knowledge to address community knowledge and practice gaps; (3) learning integration and reinforcement through the communication of action messages; and 4) community actions and mentoring support (14). A range of active learning methods, including posters, pictures, storytelling, role-plays, provocative questioning, and personal reflections, were used in the Community Conversations. The use of illustrations such as posters and pictures encouraged the active participation of community members and provided a structure to guide the conversations.

We formed a team of local facilitators comprising research and development partners who have familiarity with the communities and speak the local languages. In the Yabello district, we worked through local translators. The local partners played key roles in contextualizing or localizing the discussion issues, facilitation, and documentation of the Community Conversations. We trained the local partners on the methodological approach, facilitation and note-taking protocols.

Based on developed criteria, together with the local partners, we selected 94 community members (42 women including married and household heads) and 16 (5 female) local service providers in the study sites. In identifying the participants, we strived for a diversity of opinions and perspectives to achieve a richer dialogue, collaborative learning, and community actions. We used single- and mixed-sex discussion groups to explore community members' gendered perceptions of animal welfare and influence their attitudes and practices toward gender-equitable animal welfare management.

The study was planned with local authorities, and they gave their approval for the work and played an active role in the implementation. Oral consent was obtained from the community participants prior to the commencement of the Community Conversations. Human ethics approval was obtained from the Institutional Research Ethics Committee of the International Livestock Research Institute (ILRI-IREC2018-10).

### Community Conversations discussion topics and questions

Engaging about 55 participants and running typically through 3–4 h in each study site, the Community Conversations explored the following open-ended discussion questions (Table 2). The topics and discussion questions were used as a checklist to guide the conversations and probing techniques were used to have a deeper understanding of the issues.

**Table 2:** Community Conversation topics and discussion questions.

**Topics**	**Discussion questions**
What is animal welfare? Why is it important?	•Can animals be happy or sad? Do they have feelings like humans? •How do you know when animals are sad or happy? •What makes animals happy or sad? Do you think animals suffer from physical pain? •Why is it important for animals to perform their natural behaviors? •What does animal welfare mean to you? What is the local term for animal welfare? •What are community members' attitudes and values toward animals? Are there any traditional customs, beliefs, or sayings about animals or their care? How are animals perceived or viewed in the community? •How do you describe good or bad animal welfare conditions in your community? Who in this community is regarded as the best animal caregiver? What makes this person the best animal caregiver? •When is moving or handling your animals easy? Does this differ by age, gender, personality, or experience of the handler? •What do you think are the effects of good and bad animal handling on the animal and the handler? •What do you think are the benefits of improving the welfare of your animals?
What are community members' animal welfare constraints, needs and options? How do these differ by gender?	•What do animals need to be healthy, happy, and productive? •What could happen if these animal needs were not met? •How do you observe or identify these effects on the animals? •What are the most common animal welfare issues that affect all species of animals in the community? •How well do you think you are meeting the needs of your animals? What are your constraints, needs, and options to improve the welfare of your animals? •What are the risks and opportunities for women and men in improving the welfare of their animals?

### Data collection and analysis

We used process documentation to collect qualitative data on the Community Conversations implementation process and outcomes. Process note-taking tools and reflection checklists were used to record conversation results, reflect on the process, summarize emerging themes, interpret results, and draw lessons, which were documented in reflective reports (29). An after-event reflection and insight-making process with the facilitation team facilitated on-the-spot analysis, interpretation, and validation of Community Conversations results and experiences.

An inductive thematic analysis (30), which involves content analysis from documents, was used to analyze data contained in the research reports and field notes. We carefully reviewed the research reports and sought for thematic patterns to establish emerging themes and key findings and illustrate these with direct quotes from community members. The themes were also complemented with the literature to add context and validity. We considered socio-cultural, demographic, and agroecological factors in making a comparative data analysis.

## Results

### Multi-dimensional understanding of animal welfare

**Figure 1:**
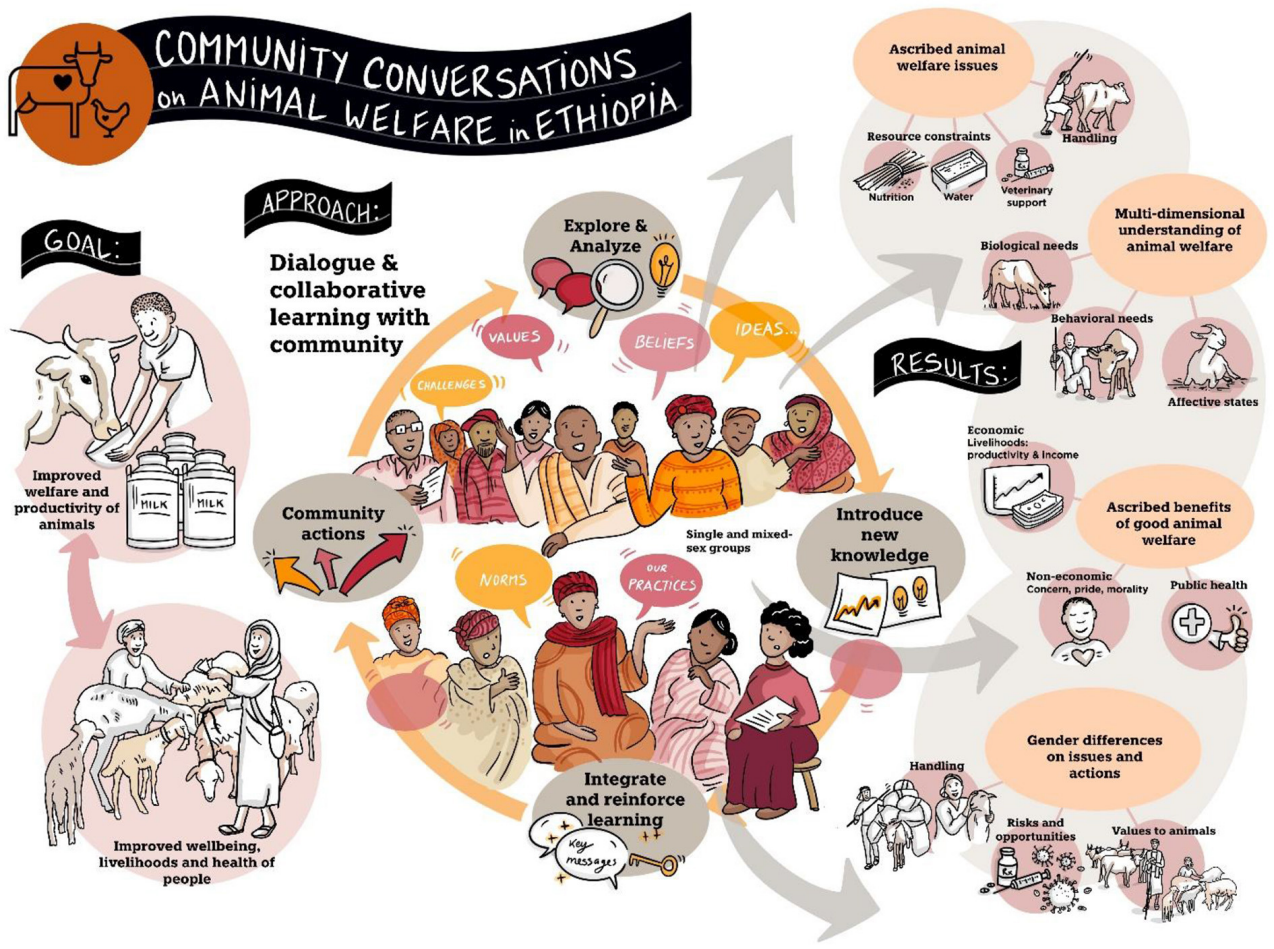
Community Conversations process and results.

In the Community Conversations, community members demonstrated a basic understanding of animal welfare. Figure 1 illustrates the Community Conversations process and the main results. Community members stated that “animals have feelings like humans” and identified the conditions in which animals can be happy or sad and the behavioral responses of animals in those conditions. They said that animals are happy during rainy seasons because they get enough feed and water. When there is rain, animals show signs of happiness like playing with each other and putting their tails up while running. They are sad during a drought season. Animals are unhappy when they are hungry, their shelter is unclean, they are sick, they get injured, or they are beaten. When they are unhappy, they have their head down, and they do not want to run and play. When they are not fed well, they do not want to go to their shelter; they want to go away, and they do not allow their offspring to suckle. A woman participant said, “animals are sad and feel bad when there is no feed and water, and when they are sick.” Community members stated that animals can suffer from diseases or physical injuries when they do not receive good care or treatment.

In the study sites, community members recognized the need for their animals to express natural behaviors. They said that when animals are tethered or kept indoors all the time, it is not good for their health and body condition. For example, in Menz Gera, a woman participant said, “they become weak.” Community members knew when their animals express the need for free movement. They said that animals show behaviors like making loud noises, becoming restless, and fighting one another. “When they are released from their shelter”, a women farmer said, “animals run freely, and they love grazing in the open”. Another woman said, “when animals get refreshed, they rest peacefully and longer in their shelters.”

In Yabello, community members described animal welfare as “fulfilling what animals need and not adversely compromising their feelings.” Similarly, in Menz Gera, community members described animal welfare as “*kibkabe*” meaning ensuring the wellbeing of animals or giving them good care. The community members described animal welfare to include having clean housing, timely feeding, leaving animals freely in the environment, not tying animals all the time, giving animals protection from predators, watering animals freely, and keeping them healthy.

In describing animal welfare, community members commonly associated feeding and health with the welfare of animals. They readily identified the biological needs of animals such as health, clean shelters, clean water, and sufficient feed. However, it was not obvious for them to identify the affective state and natural behavior of animals. These components of animal welfare did not come to their mind at first. It was through follow-up probing questions that they started to recognize these components of animal welfare.

Through the Community Conversations, community members described good and bad animal welfare conditions and assessed their own existing animal management practices. They said that the welfare of their animals is affected during drought due to a shortage of feed and water. They described bad animal welfare as keeping animals in dirty housing, withholding treatment, and disturbing animals by beating or yelling at them. The community members believed that animals need clean and comfortable shelter. They said that “animals refuse to enter unclean and wet shelters, and they rest for a shorter time in uncomfortable shelters.” While recognizing good animal welfare conditions, community members also identified their limitations in giving good care to their animals, mainly related to resource constraints and handling behaviors.

### Community attitudes and values toward animals

In the study sites, livestock is the main source of livelihood, social status, and prestige. Community members stated that they value their animals as they have no other options for living rather than their animals. In Yabello, women community members said, “our animals are many things for us. Cows give us milk, and bulls are used for plowing. Camels and donkeys are used for transportation. Sheep and goats are income sources to purchase household consumables.”

In Menz Gera, community members said, “the existence of an animal is its owner” meaning it is the owner who provides care and protection to the animal. From cultural and religious perspectives, they argued that “it is a sin not to give care for animals”, and they believed that “animal cruelty can cause judgment in heaven”.

In Yabello, community members demonstrated closer connection and proximity with their animals. During the Community Conversations, they explained that they understand well their animals and express feelings about them, especially cattle. They stated that animals know their names and follow instructions from their owner or associated family member. Women and men community members used songs to communicate and connect with their animals. Women sang for their animals, especially dairy cows, a song called “*sirba*”, welcoming them in the evening and facilitating milking. The song conveys the importance of animals as the source of subsistence for the household. It literally means that women give birth to children, and animals feed the children to grow. Similarly, male community members sang for their animals, especially cattle, a song called “*weedduu*” during plowing farm plots or when herding animals. The songs signify the importance of animals in the social status or prestige of male community members. In addition, the songs show the connections animal owners have with their animals. Expressing feelings about their animals using songs have a positive impact on the affective state of both the owners and the animals. The songs portray positive attitudes of animal owners to their animals, which is associated with more humane behavior toward the animals.

The songs also showed that women and men community members attached different values or meanings to animals depending on the purpose of the animals and their relationships with the animals. Community members gave more value and care to cattle, especially plowing oxen and milking cows, followed by small ruminants. As women focus on the provision of food and household wellbeing, they value and have a closer relationship with dairy cows, while men focus on social status or prestige, and thus attach more value to cattle. While pack animals such as donkeys play a key role in a rural economy, they received lower levels of welfare.

Although community members knew the value of animals, there was limited knowledge of what their animals needed to experience good welfare. When it comes to good animal management practice, there were limitations both due to resource constraints, lack of knowledge, and behavior of owners or caregivers. Their knowledge of diseases and the actual care they give to animals in terms of preventive measures was limited. There was also a knowledge gap regarding nutrition, behavioral and health problems of animals.

### Community perceptions of human-animal relationships

While community members expressed positive attitudes and values toward animals, they also identified gaps in handling and giving good care to their animals. In Menz Gera, men and boys were reported to hit and yell at animals. A woman participant narrated that once her son tied up the legs of a sheep and beat it. Another woman said, “when I was driving my loaded donkey to a milling house, it refused to go. I requested a man to help me move the donkey. He beat it harshly, but the donkey did not move. Then he stopped beating the donkey and said he was sorry for my husband who has to handle the donkey”.

On the contrary, in Menz Gera, women were reported to handle animals in a calm and friendly manner. Women participants explained that animals learn and develop fear if they are beaten or yelled at. A woman participant said, “animals run away, stop or change the direction of movement when they hear the voice of children or male members of the household.” Animals know who is taking good care of them, and they make noise when they see the caregiver or hear their voices. A male participant said, “animals behave like children. If I treat my son positively, he will call me father and approach me affectionately. Likewise, animals also know who gives them good care and express their connection with the handler.”

Community members believed that animals should be handled properly. In Menz Gera, a male participant stated that “if we force and handle animals harshly, they will not move, they can be injured, or they could kick the handler.” They explained that good handling is not only beneficial for the animals but also for the handler. When animals are handled badly, they can be aggressive, difficult to handle, and can injure the handler by kicking or biting. Bad handling of animals also affects the emotion of handlers. A male participant said, “I feel guilty when animals experience physical pain due to bad handling.”

In Yabello, community members reported that they handled their animals calmly and never used force. They called their animals by name, restrained them by a rope, and showed them friendly behavior for easy handling. Community members reported that they never beat their animals harshly and do not yell at them. They indicated that they use different physical restraining techniques to manage fearful, strong, and aggressive animals.

In Menz Gera, although community members described donkeys as “beating tolerating animals”, they believed that “all animals can feel physical pain as humans do and become unhappy or frustrated when they are harshly beaten.” A woman participant said, “it is only stone that does not feel pain.” Animals can become fearful and want to run away from humans when they are shouted at. “When animals experience physical pain or are worked hard”, community members said, “they become fearful, have stripes on their skin when beaten, bend their bodies, fall on the ground, and do not move.”

Male participants reported that ox beating during plowing was common, and if the ox were lazy, the beating was harsh. They even became aggressive when beating unresponsive animals. They said, “though we know that oxen feel physical pain, our focus is on finishing the plowing.” A participant said, “it is the sunset which sets the oxen free”, meaning the oxen are overworked throughout the day, especially during planting seasons. Another participant said, “a farmer who missed plowing in September cannot recover in September of the next year”, meaning the month of September is a peak plowing season. After plowing, farmers said, “we massage the skin lesions or strips on the oxen and provide feed and water, but the oxen refuse to eat or drink, and this makes us feel bad.”

While community members described the behavioral responses of animals due to negative handling, they were not aware of how negative handling can affect the health, growth, and productivity of their animals. Through the Community Conversations, community members recognized the effect of good and bad animal handling on the affective state, health, and productivity of their animals. They understood that animals need safety and relaxation in their handling and expressed commitment to handle their animals by gentle instruction rather than by beating the animals.

### Ascribed benefits of good animal welfare

The Community Conversations showed that community members had a good understanding of the relationship between animal welfare and their livelihoods. Community members stated that their livelihoods depend on animals and the animals also depend on their owners. Describing this reciprocal effect, a male farmer said, “to benefit from animals, we have to take care of them. It is a give-and-take relationship”. The community members also recognized the relationship between animal welfare and productivity. They stated that “when our animals are kept in good condition and are not stressed, they behave well and become productive. From our cows, we get good milk; healthy and strong bulls plow the land well”. A woman participant said, “when milking I calmly handle my cow calling her by name and massaging her rather than beating or yelling at her. This way, my cow stops by herself for milking and gives more milk (does not withhold the milk). Also, when I keep her house clean, I get hygienic milk”. A farmer in Menz Gera said, “keeping animals in good condition will save treatment costs”.

Community members also realized the public health benefits of improving animal welfare. They argued that keeping their animals healthy and in good condition also means keeping their household members in good health and well-being. A woman participant in Menz Gera said, “if animal shelters are not clean and dry, a bad smell can cause respiratory infections in humans”.

Community members also described the non-economic benefits of good animal welfare. They said that it is ethical and morally satisfying to give good care to animals. They felt guilty when animals experience physical pain and suffer from diseases or injuries. The community expressions about animal caregiving and handling showed how good animal welfare is important for their emotional well-being. In the study sites, community members stated that they become happy and feel better when they give good care to their animals. This close association and inter-dependence between animal welfare, livelihoods, and public health is an important reminder of how good animal welfare has both productivity as well as non-economic benefits for animal owners.

### Ascribed animal welfare issues

During Community Conversations, community members identified the needs of their animals and the constraints to meet those needs. A woman participant said, “if it is not for speaking, animals have similar needs and feelings as humans”. Other participants said that “it is not only humans who need good things; animals also need good things”. Animals need prevention and control of diseases, safe grazing, and control of parasite infections.

Women and men community members identified feed, salt, water, animal health, housing, and animal handling as common animal welfare issues. Describing the importance of sanitation (keeping animal shelters clean and dry), a male participant in Menz Gera said, “if you see dirty fleece, you can tell the sanitation in animal shelters”. Similarly, a woman participant said, “the smell of the sheep can indicate the sanitation condition of animal shelters”. Through the Community Conversations, community members recognized the consequences of poor animal welfare conditions. They explained that “when animal shelters are not clean, they can cause infections.” They also indicated that “if animals do not get adequate feed, they will be emaciated, do not give enough milk and cannot resist or are susceptible to infectious diseases.”

Community members indicated that they could observe animal behavior related to environmental conditions and “hear the voices” of their animals. They said that animals show behavioral responses such as reduced activity and responsiveness. Tail biting in dogs, vocalization of animals, running or unusual behaviors and feather pecking in poultry are behaviors induced by environmental inadequacies. These abnormal behavior patterns reflect inadequacies of the animal's environment or bad animal keeper behavior. Community members explained that behavioral observations related to feeding, drinking, or resting can give insights into the animal's feelings and requirements. They said that animals that are discomforted due to poor housing conditions, such as standing all night, show signs of injuries to their legs like staggering, stopping with one leg, or incoordination. Sick animals reduce their body weight. Fearful animals stay alarmed, run to other animals, or stand when approached.

Community members identified constraints to improving the welfare of their animals related to feeding, water, veterinary drugs, and service provision. They also described situations where animal handling could be improved. They stated that the shortage of feed and water critically affected the welfare of their animals. In Yabello, a male participant said, “we drive animals long distances on rough terrain in search of feed and water, which makes them exhausted or injured.” Another participant said, “our animals get water in an interval of 2 or 3 days”. Health-related constraints of animal welfare were the lack of veterinary clinics, veterinary drugs, and trained animal health workers. Community members indicated that the veterinary clinic was far from their village. The animal health workers were also not available all the time in the local veterinary clinic. Community members reported that animal health workers lived in town, and they were not accessible as they needed them. The veterinary clinic also lacked essential drugs and vaccines. As a result, community members often buy veterinary drugs from the market and administer the drugs by themselves or community animal health workers. They also indicated that vaccinations for common diseases were not available for all animal species, especially camels and equines.

### Community actions

The Community Conversations aimed to not only identify and analyze animal welfare issues but also encourage community members to develop practical strategies to solve the issues along with local service providers. The community members set their vision for improved animal welfare and the actions that they thought should be taken (Table 3). The community action plans can contribute to improved human and animal welfare. Through Community Conversations, local service providers understood community issues and the community actions informed local planning processes, which can improve the capacity of both community members and local service providers to take actions toward improving the welfare of animals.

**Table 3:** Community actions to improve animal welfare.

**Priority animal welfare issues**	**Community actions**	**Expected benefits**
Feed and water availability	•Timely collection of grass/haymaking/crop residues •Reduce herd size •Introduce improved forage production •Improve feeding and watering troughs •Improve grazing land management practices •Improve ration formulation of locally available feed resources •Pond construction and fencing for water points	•Increased feed and water availability •Happy, healthy, and productive animals •Saving animals' lives •Animals gain body weight •Good milk and butter production
Animal health management	•Regular vaccination and deworming •Construct animal health posts •Community mobilization based on scheduled vaccination/deworming programs •Improve animal housing sanitation by frequently cleaning barns •Buy veterinary drugs from approved sources •Monitor body and health condition of animals •Consult veterinarians when animals are sick •Report disease outbreaks timely	•Healthy, happy, and productive animals •Reduced cost of animal treatment •Reduced transmission of diseases to humans •Reduced effect of drug resistance
Animal handling practices	•Teach children not to hit animals •Hold household discussions about the effect of bad animal handling on the feelings and productivity of animals	•Happy and productive animals •Satisfaction of handlers

The local partners found the community dialogues engaging and empowering. The conversations helped create shared understanding (beyond individual learning) through social interaction and collaborative learning among community members and local service providers leading to the implementation of joint actions.

Innovative approaches such as putting women drawn from communities at the heart of animal welfare will achieve better results. The Community Conversations encouraged women and men community members to take ownership of animal welfare challenges and discuss solutions and think through their implementation, articulating the changes that they are likely to make. In Menz Gera, community members stated that the Community Conversations gave them a better understanding of animal welfare issues, and what it takes to meet the welfare of their animals. Both women and men community members recognized the importance of meeting the needs of their animals. A woman participant said, “the community discussions expanded our understanding of animal welfare issues”.

## Discussion

Through the Community Conversations, community members gained a multi-dimensional understanding of animal welfare. They described animal welfare as satisfying the biological functioning of animals, such as feed, water, shelter, and health care. However, it was not automatic for community members to identify the affective states and natural behaviors of animals in their view of animal welfare. Upon further in-depth discussion, community members recognized the feelings and natural behavior of animals as animal welfare components. Similarly, based on a semi-systematic review and thematic analysis of factors that influence farmers' views on farm animal welfare, Balzani and Hanlon (6) described three farmer categories according to their views on animal welfare. They showed that the biological functioning of an animal was the most common view of farmers, the affective state of an animal emerged as the second most common view, and the third category related to the ability of an animal to engage in natural behavior.

Community members also explored multiple benefits of good animal welfare, which are the drivers for their actions to improve the welfare of their animals. They described the welfare of their animals as being intertwined with their own livelihoods. While community members pronounced more on the economic benefits of good animal welfare (such as improved productivity of animals, saving on health costs, and increased incomes), they also acknowledged the non-economic benefits of good animal welfare (such as public health and psychological wellbeing of people). However, community members mostly described what the animal owners could benefit from good animal welfare, and they did not mention the benefits to the animals themselves. Similarly, using focus group discussions, Sinclair et al. (31) showed that economic and public health reasons (such as productivity, meat quality, food safety, human health, and livelihoods) were the most mentioned benefits of good animal welfare among livestock stakeholders across Asia and that improving animal welfare in the benefit of the animals themselves was not reported in most of the study countries.

The study shows that gender, age, and experience of animal owners seem to influence how they handle their animals. Previous studies (32–34) also found that individual characteristics such as the age and experience of the handler and cultural variables could influence human attitudes to animals and their welfare. Bad animal handling can cause stress and aggression both in animals and handlers. Hemsworth (35) showed that negative animal handling, such as beating animals harshly, shouting, and rapid movement, can make animals fearful, stressed, and unhappy. This can affect the health, welfare, and productivity of animals (13, 36). While farmers and pastoralists can recognize visible behavioral responses of their animals to negative handlings, such as animals showing fear, avoidance of humans, and refusing to feed, they may not recognize the psychological and physiological effects of negative handing on animals and their health, growth, productivity, and welfare. Through the conversations, community members recognized that good animal handling is as important as meeting the biological needs of their animals. The expressions community members used in describing animal welfare show that they have a sense of empathy for animals and their sense of responsibility and moral obligation for the good caregiving of their animals.

While animal beating is common by men and boys in highland areas (Menz Gera), in the pastoralist communities (Yabello), men reported good animal handling practices. This can be because of differences in the value systems, religious and cultural beliefs, and production systems of the communities. Animals, such as cattle, were more valued for production purposes and animal handling was problematic in Menz Gera, for example, handling of plowing oxen. In contrast, social status or prestige was more important to pastoralists and animal handling was much better in Yabello. This was demonstrated in the songs community members sing their animals that express their values and relationships with their animals. This proximity of pastoralist communities with their animals like massaging, speaking to animals (calling animals by names), and singing songs to animals fosters empathy and is at the foundation of their understanding of animal welfare (6).

In the study sites, women tended to have more positive attitudes toward animals and are sensitive to the way they handle animals. This may be due to gender differences in empathy and values to animals (3). This may also be because women frequently handle milking cows (32) and develop more attachment to these animals than men, who frequently handle plowing oxen. Similarly, Campler et al. (33) showed that empathy attribute-related questions positively correlate with the gender of animal caretakers.

The understanding of community members' gendered perceptions of animal welfare and values for animals is important to inform gender-responsive animal welfare interventions. However, gender biases may be limiting service delivery and knowledge sharing both at the livestock extension service and community levels. While women have more positive attitudes to animals and animal welfare, gender norms and practices may limit their decision-making role in welfare improvement (21). Gender transformative approaches, such as Community Conversations, can support efforts to achieve both gender equality and animal welfare outcomes (37). The use of both single- and mixed-sex groups in the Community Conversations helped challenge community perceptions and influence their attitudes toward gender-equitable animal welfare management. Similarly, Lemma et al. (38) and Mulema et al. (22) showed that Community Conversations are supporting gender equality efforts in Ethiopia.

Understanding the constraints, risks, and opportunities of rural communities and the needs of the animals they care for can help improve both livelihoods and animal welfare outcomes. Given their gender roles in livestock management, women and men community members may have different risks and opportunities for improving the welfare of their animals and their own livelihoods. Women may be more exposed to zoonotic diseases (22, 39) and can be physically injured in handling animals. Animal owners' attitudes toward animals, their knowledge about giving care to animals, and resource and service constraints can limit their ability to improve the welfare of their animals (36). Animal welfare constraints are more prominent in small-scale and pastoralist farming systems, such as Yabello, where access to resources and livestock services is limited.

While community members demonstrate good knowledge of animal welfare and can identify where improvements could be made, there is a gap when it comes to addressing these issues. This gap extends to the veterinary support services that work with the farmers and pastoralists. As primary animal caregivers, community members need advice and training support to expand their knowledge and skills based on an understanding of their animal welfare perceptions, constraints, and needs (5, 40). This study and previous studies (22, 23) showed that the Community Conversations approach proved effective in strengthening the capacity of community members and local service providers to improve the welfare of animals in a gender-responsive manner.

## Conclusion

The Community Conversations enabled community members and local service providers to better understand the multi-dimensional issues around animal welfare and how this can influence welfare improvement interventions. Community members described animal welfare as focusing on the biological needs of animals such as feed, water, and health, but there was also a good acknowledgment of the behavioral needs of animals as well as their ability to experience affective states. The community members identified feed, animal health, sanitation, and animal handling as priority animal welfare issues. There were also limitations in meeting the needs of animals both due to resource constraints, lack of knowledge, limited livestock services, and behavior of owners or caregivers. Changing the attitudes and practices of community members is critical for improving the welfare of their animals and their own livelihoods.

## Data availability statement

The datasets presented in this study can be found in online repositories. The names of the repository/repositories and accession number(s) can be found in the article/supplementary material.

## Ethics statement

The studies involving human participants were reviewed and approved by the Institutional Research Ethics Committee of the International Livestock Research Institute. Written informed consent for participation was not required for this study in accordance with the national legislation and the institutional requirements.

## Author contributions

The concept of the Community Conversations on animal welfare was developed by ML, RD, and BW. Fieldwork was conducted by ML, MM, and AK. A first draft of the manuscript was prepared by ML, expanded by RD, BW, and GA, then further revised by ML. All authors reviewed and approved the final version.

## Funding

This research was conducted as part of the CGIAR Research Program on Livestock and was supported by contributors to the CGIAR Trust Fund. CGIAR is a global research partnership for a food-secure future. Its science is carried out by 15 Research Centers in close collaboration with hundreds of partners across the globe www.cgiar.org.

## Conflict of interest

The authors declare that the research was conducted in the absence of any commercial or financial relationships that could be construed as a potential conflict of interest.

## Publisher's note

All claims expressed in this article are solely those of the authors and do not necessarily represent those of their affiliated organizations, or those of the publisher, the editors and the reviewers. Any product that may be evaluated in this article, or claim that may be made by its manufacturer, is not guaranteed or endorsed by the publisher.

## References

[1] Fraser D Duncan IJ Edwards SA Grandin T Gregory NG Guyonnet V. General principles for the welfare of animals in production systems: the underlying science and its application. Vet J. (2013) 198:19–27. 10.1016/j.tvjl.2013.06.028 23899406

[2] Fraser D Weary DM Pajor EA Milligan BN. A scientific conception of animal welfare that reflects ethical concerns. Anim Welf. (1997) 6:187–205.

[3] Cornish A Raubenheimer D McGreevy P. What we know about the public's level of concern for farm animal welfare in food production in developed countries. Animals. (2016) 6:74. 10.3390/ani6110074 27854336 PMC5126776

[4] Weary DM Robbins JA. Understanding the multiple conceptions of animal welfare. Anim Welf. (2019) 28:33–40. 10.7120/09627286.28.1.033

[5] Fuente SA Caselli CB Schiel N. People's perception on animal welfare: why does it matter? Ethnobio Conserv. (2017) 6:18. 10.15451/ec2017106.1817

[6] Balzani A Hanlon A. Factors that influence farmers' views on farm animal welfare: a semi-systematic review and thematic analysis. Animals. (2020) 10:1524. 10.3390/ani10091524 32872206 PMC7552314

[7] Doyle RE Wieland B Saville K Grace D Campbell AJD. The importance of animal welfare and Veterinary Services in a changing world. Rev Sci Tech. (2021) 40:469–81. 10.20506/rst.40.2.3238 34542100

[8] Global Alliance for Improved Nutrition. Consumer Vendor Perspectives on Practices Related to Food Safety in Ethiopia: A Review. (2022). A USAID EatSafe Project Report. Available online at: https://pdf.usaid.gov/pdf_docs/PA00Z882.pdf (accessed May 9, 2022).

[9] Keeling L Tunón H Olmos AG Berg C Jones M Stuardo L. Animal welfare and the United Nations sustainable development goals. Front Vet Sci. (2019) 6:336. 10.3389/fvets.2019.00336 31649940 PMC6797006

[10] FAO. Livestock, Health, Livelihoods, and the Environment in Ethiopia. An Integrated Analysis. (2019). Rome: FAO.

[11] Asebe G Gelayenew B Kumar A. The general status of animal welfare in developing countries: the case of Ethiopia. J Veterinary Sci Techno. (2016) 7:3. 10.4172/2157-7579.1000332

[12] Kauppinen T Vainio A Valros A Rita H Vesala KM. Improving animal welfare: qualitative and quantitative methodology in the study of farmers' attitudes. Animal Welfare. (2010) 19:523–36.

[13] Jerlström J. Animal Welfare in Ethiopia: Transport to and Handling of Cattle at Markets in Addis Ababa and Ambo. Degree Project in Animal Science. Uppsala: Swedish University of Agricultural Sciences (SLU) (2013).

[14] Lemma M Mekonnen M Tigabie A. Community Conversation: An Approach for Collaborative Learning and Action in Animal Health Management. Nairobi: ILRI (2021).

[15] Central Statistical Agency [Ethiopia] and ORC Macro. Ethiopia Demographic and Health Survey 2005. Addis Ababa, Ethiopia and Calverton, Maryland: Central Statistical Agency and ORC Macro (2006).

[16] Angassa A Oba G. Herder perceptions on impacts of range enclosures, crop farming, fire ban and bush encroachment on the rangelands of Borana, southern Ethiopia. Hum Ecol. (2008) 36:201–15. 10.1007/s10745-007-9156-z

[17] Wako G Tadesse M Angassa A. Camel management as an adaptive strategy to climate change by pastoralists in southern Ethiopia. Ecol Proc. (2017) 6:26. 10.1186/s13717-017-0093-5

[18] Shapiro BI Gebru G Desta S Negassa A Nigussie K Aboset G Mechale H. Ethiopia Livestock Sector Analysis: A 15-Year Livestock Sector Strategy. ILRI Project Report. Nairobi: ILRI (2017).

[19] FAO. The Future of Livestock in Ethiopia. Opportunities and Challenges in the Face of Uncertainty. Rome: FAO (2019).

[20] Kinati W Mulema A. Gender issues in livestock production systems in Ethiopia: A literature review. J Livestock Sci. (2019) 10:66–80. 10.33259/JLivestSci.2019.66-80

[21] Lemma M Gizaw S Etafa A Mulema AA Wieland B. Gender Integration in the Ethiopian Agricultural Extension System: A Literature Review. Nairobi: ILRI (2020).

[22] Mulema AA Kinati W Lemma M Mekonnen M Alemu BG Elias B. Clapping with two hands: transforming gender relations and zoonotic disease risks through community conversations in rural Ethiopia. Hum Ecol. (2020) 48:651–63. 10.1007/s10745-020-00184-y

[23] Lemma M Kinati W Mulema AA Mekonnen M Wieland B. Community Conversations: A Community-Based Approach to Transform Gender Relations and Reduce Zoonotic Disease Risks. Nairobi: ILRI (2019).

[24] Wals AEJ van der Hoeven EMMM Blanken H. The Acoustics of Social Learning: Designing Learning Processes That Contribute to a More Sustainable World. Wageningen: Wageningen Academic Publishers (2009).

[25] Biggs S Matsaert H. Strengthening Poverty Reduction Programs Using an Actor-Oriented Approach: Examples From Natural Resources Innovation Systems. Network Paper No.134. ODI: Agricultural Research Extension Network (2004). Available online at: https://cdn.odi.org/media/documents/8260.pdf (accessed May 12, 2022).

[26] German L Stroud A. A framework for the integration of diverse learning approaches: operationalizing agricultural research and development (R&D) linkages in Eastern Africa. World Dev. (2007) 35:792–814. 10.1016/j.worlddev.2006.09.013

[27] MacDonald C. Understanding participatory action research: a qualitative research methodology option. Can J Action Res. (2012) 13:34–50. 10.33524/cjar.v13i2.37

[28] Doyle R Lemma M Mulema A Wieland B Mekonnen M. Community Conversation on Animal Welfare: A Guide to Facilitators. Nairobi: ILRI (2019).

[29] Lemma M Mekonnen M Kumbe A Demeke Y Wieland B Doyle R. Community Conversations Report on Animal Welfare. Nairobi: ILRI (2019).

[30] Nowell LS Norris JM White DE Moules NJ. Thematic analysis: striving to meet the trustworthiness criteria. Int J Qual Methods. (2017) 16:1–13. 10.1177/1609406917733847

[31] Sinclair M Fryer C Phillips CJC. The benefits of improving animal welfare from the perspective of livestock stakeholders across Asia. Animals. (2019) 9:123. 10.3390/ani9040123 30925747 PMC6524158

[32] Garsow AV Biondi MR Kowalcyk BB Vipham JL Kovac J Amenu K. Exploring the relationship between gender and food safety risks in the dairy value chain in Ethiopia. Int Dairy J. (2022) 124:105173. 10.1016/j.idairyj.2021.105173

[33] Campler MR Pairis-Garcia MD Rault JL Coleman G Arruda AG. Caretaker attitudes toward swine euthanasia. Transl Anim Sci. (2018) 2:254–62. 10.1093/tas/txy015 32704709 PMC7200551

[34] Serpell JA. Factors influencing human attitudes to animals and their welfare. Anim Welf. (2004) 13:145–51.

[35] Hemsworth P. Human-animal interactions in livestock production. Appl Anim Behav Sci. (2003) 81:185–98. 10.1016/S0168-1591(02)00280-0

[36] Martínez GM Suárez VH Ghezzi MD. Influence of the human-animal relationship on productivity and animal welfare in dairy farms. Dairy and Vet Sci J. (2019) 11:555825. 10.19080/JDVS.2019.11.55582532147267

[37] Drucza K Abebe W. Gender Transformative Methodologies in Ethiopia's Agricultural Sector: A Review. Addis Ababa: CIMMYT (2017).

[38] Lemma M Kinati W Tigabie A Mekonnen M. Community Conversations Empower Women and Transform Gender Relations in Rural Ethiopia. Innovation Brief. Addis Ababa: ICARDA (2021).

[39] Wieland B Alemu B Desta H Kinati W Mulema AA. Participatory Epidemiology Gender Analysis to Address Small Ruminant Disease Constraints in LIVESTOCK Fish AfricaRISING Project Sites in Ethiopia. (2016). Available online at: https://cgspace.cgiar.org/bitstream/handle/10568/76685/LF_peg_jul2016.pdf (accessed June 3, 2022).

[40] Atkinson O. Communication in farm animal practice 1. Farmer–vet relationships. In Pract. (2010) 32:114–7. 10.1136/inp.c836

